# A systematic review on the risk of neurodegenerative diseases and neurocognitive disorders in professional and varsity athletes

**DOI:** 10.1007/s10072-022-06319-x

**Published:** 2022-08-17

**Authors:** G. Bellomo, P. Piscopo, M. Corbo, E. Pupillo, G. Stipa, E. Beghi, N. Vanacore, E. Lacorte

**Affiliations:** 1 National Center for Disease Prevention and Health Promotion, Italian National Institute of Health, Rome, Italy; 2 Department of Neurosciences, Italian National Institute of Health, Rome, Italy; 3 Department of Neurorehabilitation Sciences, Casa Cura Policlinico, Milan, Italy; 4 Istituto Di Ricerche Farmacologiche Mario Negri IRCCS, Milan, Italy; 5 Clinical Neurophysiology Division, Neuroscience Department, S. Maria University Hospital, Terni, Italy

**Keywords:** Systematic review, Neurodegenerative diseases, Risk factors, Athletes, Clinical epidemiology

## Abstract

**Objective:**

The aim of this systematic review (SR) was to gather all available epidemiological evidence on former participation in any type of sport, at a professional and varsity level, as a potential risk factor for neurodegenerative diseases (NDs) and neurocognitive disorders (NCDs).

**Design:**

Systematic searches were performed on PubMed, the Cochrane databases, and the ISI Web of Knowledge databases. Included studies were assessed using the NOS checklist.

**Eligibility criteria for selecting studies:**

All epidemiological studies reporting data on the possible association between a clinical diagnosis of amyotrophic lateral sclerosis (ALS)/motor neuron disease (MND), dementia or mild cognitive impairment (MCI), Parkinson’s disease (PD), chronic traumatic encephalopathy (CTE) at any stage and with any clinical pattern and the former participation in any types of sport at a varsity and professional level were included.

**Results:**

Data from the 17 included studies showed a higher frequency of NDs and NCDs in former soccer and American football players. Updating the previous SR confirmed a higher frequency of ALS/MND in former soccer players. Data reported a significantly higher risk of dementia/AD in former soccer players, and of MCI in former American football players. Results also showed a significantly higher risk of PD in former soccer and American football players, and a significantly higher risk of CTE in former boxers and American football players.

**Summary/conclusions:**

This SR confirmed a higher risk of NDs and NCDs in former professional/varsity athletes. However, the pathological mechanisms underlying this association remain unclear, and further high-quality studies should be performed to clarify whether the association could be sport specific.

## Background

Previous epidemiological studies reported an association between professional sport and neurodegenerative diseases (NDs) and neurocognitive disorders (NCDs). These include a range of conditions primarily affecting brain and central nervous system (CNS) functioning [1, 2]. NDs are multifactorial, still incurable, and debilitating conditions resulting in the progressive degeneration and/or death of nerve cells [1]. The pathophysiological mechanisms underlying these conditions can differ, causing deficits ranging from memory loss and cognitive impairment to movement and gait deficits and impairments in language and breathing abilities [3]. Amyotrophic lateral sclerosis (ALS) represents the most common motor neuron disease (MND) and described as a rare progressive neuromuscular disease, typically causes the degeneration of both the upper and lower motor neurons but may also include cognitive and behavioral symptoms [4–6]. Parkinson’s disease (PD) is primarily characterized by a specific set of motor symptoms, including tremor, rigidity, and bradykinesia, and by an impairment of balance and postural reflexes. It can also cause several non-motor symptoms, such as gastrointestinal and urogenital disorders, and psychiatric symptoms, including depression [7]. NCDs is a broad term for defining several different chronic and progressive conditions affecting the ability to think, remember and make decisions. Symptoms may vary according to the type and severity of disease, and include memory loss, attention and communication deficits, and impairments in the ability to reason and solve problems [8, 9]. According to the DSM-5 dementia and mild NCD (MCI) are the main categories of NCDs. As for dementia, some subtypes are included among NDs, as they are caused by neurodegenerative mechanisms, while some types of dementia and other NCDs can be due to other causes, including secondary causes as intoxication or trauma [2]. The most common cause of ND dementia is Alzheimer's disease (AD), which is characterized by memory loss and specific deficits in language, sensory, motor and/or executive functions [8, 10]. Among the risk factors for dementia, the most known are genetic factors and aging. However, gender, educational level, and some health conditions are also included among risk factors [11].

Some studies investigated previous participation in some contact sports as a potential risk factor for neurodegenerative diseases (ND) and neurocognitive disorders (NCD) [12–14] focusing on pathological changes in former players [15, 16] in particular, soccer [14, 17–21] and American football players [12, 13]. Some small studies and reports have suggested that sports involving repeated traumatic brain injuries (TBI) may cause a long-term risk of NDs [22]. When considering specific NDs or NCDs, a relatively recent systematic review reported a significantly higher frequency of ALS in former professional and varsity soccer, and American football players [23]. The most frequently investigated potential sport-related risk factors for ALS and for all other NDs and NCDs, include multiple trauma and concussion [24, 25] along with oxidative stress [26] and exposure to toxic substances/chemicals (e.g., pesticides) [27, 28] or drugs/dietary supplements [28]. However, as for ALS, a more recent hypothesis also suggested that former athletes might share a phenotype causing simultaneously a higher athletic predisposition, with lower body mass index (BMI) and cardiovascular risk and higher physical fitness, and a higher risk of ALS [28, 29]. Another relatively recent systematic review, specifically focusing on long-term consequences of repeated concussions, reported that former participants in contact, collision, and combat sports seem to be at an increased risk of cognitive impairment and other mental health problems [30]. The review reported a higher frequency of cognitive decline and some NCDs and NDs in former professional athletes, considering NDs/NCDs along with diseases of the nervous system and sense organs, and other mental health conditions, including suicide and cognitive decline, hypothesizing multiple trauma and head injuries as potential causes of such condition. Multiple concussion and trauma and their potential role in the long-term onset of NDs or NCDs have been widely investigated, and are still a matter of debate, particularly in relation to popular sports such as soccer and rugby [31]. However, even though repeated head trauma has been linked to irreversible cognitive deficits [32–34] the pathophysiological mechanisms underlying this potential association are still to be clarified.

Traditionally, multiple concussion and head trauma had been known to cause the so-called ‘‘punch-drunk syndrome’’ firstly descripted in 1928 in retired boxers [35] which was associated to a basic lesion due to traumatic multiple hemorrhages, and replaced few years later by the wording ‘‘Dementia pugilistica’’ term coined in 1937 and which took into consideration in its definition the hypothesis of a type of dementia induced by brain trauma [36]. In the following decades, the scientific community focused the attention on the possible association between this type of sport and NDs, suggesting an etiological link between exposure to repeated head trauma and concussion and dementia and/or PD, even hypothesizing synergistic effects between head trauma and apolipoprotein-epsilon four in patients with Alzheimer's disease [37, 38]. In recent times, although the debate is still open, a study investigated the association between the former participation in contact and collision sports such as American football, boxing, soccer, rugby, and hockey, and a higher risk of chronic traumatic encephalopathy (CTE), highlighting a strong dose–response relationship between the duration of American football played and this pathology [39].

Based on all these hypotheses, evidence seems to suggest an association between former participation in professional sports, and mainly in contact and collision sports, with a higher risk of NDs or NCDs. Therefore, the aim of this systematic review (SR) was to gather all available epidemiological evidence on former participation in any type of sport, at a professional and varsity level, as a potential risk factor for NDs or NCDs.

## Materials and methods

This SR was performed following the methodology of the Cochrane handbook for SRs [40] and reported based on the PRISMA statement for reporting SRs and meta-analyses [41]. Systematic searches for all available literature up to February 1, 2022, were performed on PubMed, the Cochrane Databases (that gives access to: Cochrane Database of Systematic Reviews, Embase, PubMed, CT.gov, ICTRP and CINAHL), and ISI Web of Knowlegde (that gives access to: Science Citation Index, Social Sciences Citation Index, Arts and Humanities Citation Index, Emerging Sources Citation Index, Conference Proceedings Citation Index–Science, Conference Proceedings Citation Index-Social Sciences and Humanities, Book Citation Index–Science, Book Citation Index–Social Sciences and Humanities, Current Chemical Reactions, and Index Chemicus). The following terms were used: ((neurodegenerative* OR neuro-degenerative* OR “amyotrophic lateral sclerosis” OR ALS OR “motor neuron disease” OR “motor neuron diseases” OR “motor-neuron disease” OR “motor-neuron diseases” OR parkinson* OR parkinsonism* OR Alzheimer* OR dementia* OR encephalopath* OR ((cognitive OR neurocognitive OR neuro-cognitive) AND (impairment* OR degenerati* OR disord*))) AND (((professional* OR competitive* OR competition* OR elite* OR varsity* OR college* OR “high school” OR “high-school”) AND (sport* OR player*)) OR athlete* OR rider* OR fighter* OR boxeur* OR boxer* OR runner* OR skier* OR rower* OR “martial art” OR “martial arts” OR “martial artist” OR “martial artists” OR “combat sport” OR “combat sports” OR “players”)). Further relevant articles were also identified by reviewing the references of considered studies. No limitations were applied for date of publication nor study design; only studies published in English were considered. Retrieved literature was screened by two independent reviewers (GB, PP), and studies were selected based on their pertinence and relevance to the topic of the review. The full texts of selected studies were assessed for inclusion by applying the following predefined eligibility criteria. Potential disagreements were resolved by discussion or, where needed, involvement of a third reviewer (EL). All case–control and cohort studies addressing the possible association between any types of sport practiced as a varsity and/or a professional athlete and the risk of NDs and/or NCDs, were included. Since studies on this topic are performed using any type of available data, including data from structured information sources, ranging from death certificates to biological tissue banks, we included all studies reporting a diagnosis of any of the considered condition based on any type of criteria, including clinical criteria and neurobiological criteria. Therefore, all studies reporting by any means (e.g., death certificates, clinical records) a clinical, neurobiological or neuropathological diagnosis of ALS or motor neuron disease (MND), dementia of any type or mild cognitive impairment (MCI), PD, CTE at any state of disease and with any clinical pattern were also included considering the wider diagnostic category. Case reports, case series, non-systematic or narrative reviews, letters, commentaries, and editorials were excluded. Only epidemiological studies (i.e., cohort and case–control) reporting data on the frequency of any of the considered NDs and/or NCDs were included.

For studies analyzing the association between professional and/or varsity sports and ALS, we updated a previously published systematic review [23] therefore only studies published from January 2016 to February 2022 were included.

Studies meeting the eligibility criteria were qualitatively assessed using the Newcastle–Ottawa Scale (NOS) for quality assessment of non-randomized studies [42]. The NOS scale is structured in 8 items assessing the adequacy of 3 major domains: selection of the study group, comparability of the groups, and ascertainment of the exposure of interest for case–control studies or of the outcome of interest for cohort studies. A maximum of 9 stars can be assigned to each assessed study, and specifically a maximum of 4 stars for the Selection domain, 2 stars for the Comparability domain, and 3 stars for either the Outcome or the Exposure domain. The potential presence of other biases and/or methodological flaws was also considered in assessing the included studies. Data were extracted using structured tables. Specific information was gathered on the type of sport considered by each study, along with how the enrolled population of athletes was defined, and on the diagnostic criteria and procedure used to define the identified cases. Moreover, for cohort studies, specific information was also extracted on the source of the reference population. Gathered information also included study design, year of publication, characteristics of the included population, definition of the exposure(s), diagnostic criteria, length of the follow-up, and results for each analysis.

## Results

Structured search on bibliographic databases yielded 11,189 articles. Two further studies were identified by reviewing the bibliographic references of included studies [43, 44]. After selection based on the pertinence and relevance to the topic of the review, 11,191 records were excluded, leaving 47 articles meeting the selection criteria. The full texts of the selected articles were assessed for inclusion, and 30 further articles were excluded as 3 were case reports [17, 19, 45], 1 review [46], 1 reported participation in sports as a leisure activity [47] 2 reported data only on an overall measure of physical activity [48, 49] 2 included only athletes who had ≥ 1 head/brain injury [50, 51] 8 did not report any measure of risk of NDs and/or NCDs [20, 52–58] and 13 were already included in the 2016 review [59–71].

**Fig. 1:**
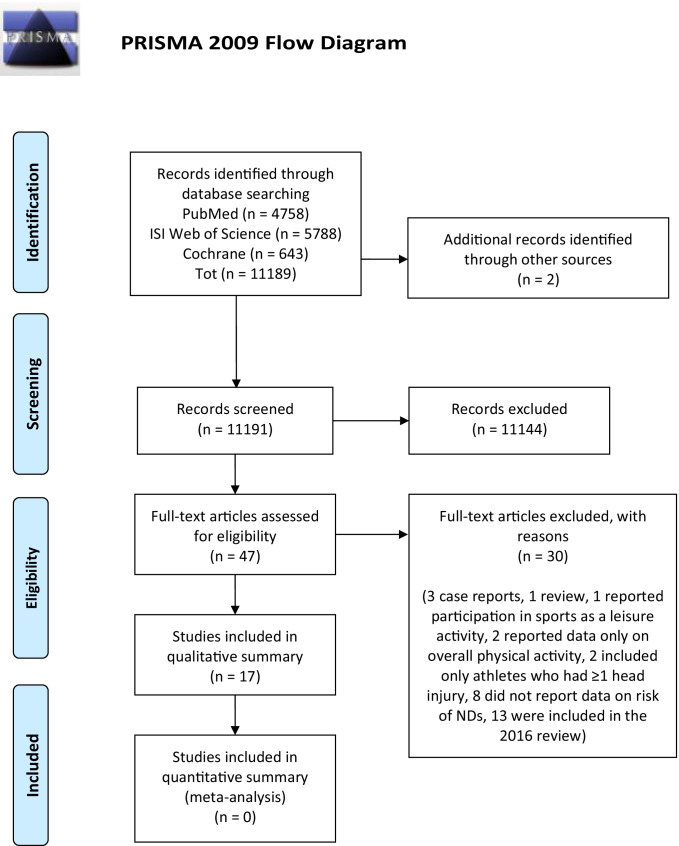
Flow diagram of literature

The flow diagram of the literature is reported in Fig. 1.

A total of 17 articles were included in the qualitative summary [43, 44, 72–86].

Overall, five cohort studies reported data on the frequency of all NDs/NCDs included in this review in former professional or varsity athletes [74, 77, 79, 82, 84]. Considering each single pathology, 10 cohort studies [82, 84] and 1 case–control study [44] reported data on the frequency of dementia/MCI in former athletes, 8 cohort studies [43, 73–75, 77, 79, 82, 84], and 1 case–control study [44] reported data on the frequency of PD or Parkinsonism, 1 cohort study [80] reported data on CTE, and 7 new cohort studies [77, 82–86] and 1 new case–control study [81] reported data on the frequency of ALS or MND. The summary of the main characteristics, quality assessment and results from the included studies were reported according to each considered outcome in Tables 1, 2, 3, 4, and 5.

**Table 1:** Summary of results from studies reporting data on the frequency of any ND and/or NCD

Characteristics					Quality assessment			Summary of findings		
								*N*. of subjects		Results
Fist author, year	Sport	Source of cases	Reference population	Exposure	Selection	Comparability	Outcome/exposure	*n*. cases among the exposed/total exposed	n. cases among the reference group/ total reference	Data ORs/HRs (95%CIs) or SIRs/PRs/SMRs/SPMRs
Cohort studies										
Russell 2021 [84]	Soccer	Hospital records, prescriptions and death certificates with ICD-9 and ICD-10 codes	Random sample of matched individuals (3:1 ratio) from the community health index database	Having been a soccer player in the Scottish League as documented in the archives of the Scottish Football Museum and individual league clubs	***	**	**	386/7676 players	366/23028 non players	Frequency of all NDs/NCDs diagnosis HR 3.66 (95% CI 2.88–4.65) *p* < 0.001
Mackay 2020 [82]	Soccer	Death certificates with ICD-9 and ICD-10 codes (primary and contributory cause), and prescriptions for dementia from electronic health records	Controls from general population matched to the players according to sex, age, and degree of social deprivation	Former professional soccer player born before January 1, 1977	**	**	**	222/7676 players	238/23028 non players	*HR for Death (95% CI) HR-U: 4.10 (2.88–5.83) < 0.001 HR-U&C: 3.53 (2.72–4.57) *p*: < 0.001
Nguyen 2019 [79]	American football baseball	Death certificates from 1979 to 2013 classified with ICD underlying (U) and underlying or contributing (U&C)	MLB cohort: from LBD of all players that appeared in ≥ 1 game at a professional level in 1871–2006	NFL cohort: from NIOSH consisting in players who played ≥ 1 season in 1959–1988	**	**	**	NFL U: 22/3419 U&C: 39/3419	MLB U: 13/2708 U&C: 16/2708	HR (95%CI) U: 2.07 (1.01–4.23) U&C: 2.99 (1.64–5.45)
Janssen 2017 [77]	American football basketball swimming wrestling	3-step procedure: screening of medical index through REP tool, review of diagnoses, diagnosis confirmed by senior neurologist	Varsity athletes (football vs non-football) enrolled in the Mayo and Rochester High Schools in 1956 to 1970	Varsity activity in football, basketball, swimming, or wrestling documented in the yearbooks	**	*	**	10/296 football players	8/190 non-football athletes	frequency all NDs/NCDs football: 10/296 (3%) non-football: 8/190 (4%)
Lehman, 2012 [74]	American football	Death certificates with ICD code for ALS in effect at death	US male mortality rates (1960–2007) for 119 cause of death categories	Having played in the National Football League for at least 5 seasons between 1959 and 1988	****	**	*	U: 10/3439 players U&C: 17/3439 players	–	All NDs/NCDs (standardized mortality rates) SMR U: 2.83 (1.36–5.21) SMR U&C: 3.26 (1.90–5.22) SMR-non-speed: 1.58 (0.33–4.61), 3 cases SMR-speed: 4.74 (2.59–7.95), 14 cases

*MLB* US Major League Baseball, *NFL* National Football League, *NIOSH* National Institute for Occupational Safety and Health, *LBD* Lahman Baseball Database, *HR* Hazard ratio, *SMR* standardized mortality ratio, *CI* confidence interval, *U* underlying, *U&C* underlying and contributing

**Table 2:** Summary of results from studies reporting data on the frequency of ALS or MND

Characteristics					Quality assessment			Summary of findings		
								*N*. of subjects		Results
Fist author, year	Sport	Source of cases	Reference population	Exposure	Selection	Comparability	Outcome/Exposure	*n*. cases among the exposed/total exposed	*n*. cases among the reference group/ total reference	Data ORs/HRs (95%CIs) or SIRs/PRs/SMRs/SPMRs
Cohort studies										
Daneshvar 2021 [86]	American football	Diagnoses and death records from news reports and/or obituaries matched to NFL records and National Death Index	Centers for Disease Control and Prevention National Institute for Occupational Safety and Health Life Table Analysis System	Having played at least 1 regular season as a professional in the NFL from 1970 to 2019	***	*	***	55/19423 players	–	SIR 3.59 (95% CI 2.58–4.93) SMR 3.94 (95% CI 2.62–5.69)
Russell 2021 [84]	Soccer	Hospital records, prescriptions and death certificates with ICD-9 and ICD-10 codes	Random sample of matched individuals (3:1 ratio) from the community health index database	Having been a soccer player in the Scottish League as documented in the archives of the Scottish Football Museum and individual league clubs	***	**	**	24/7676 players	23/23028 non-players	frequency of MND diagnosis HR 3.52 (95% CI 1.81–6.88) *p < *0.001
Gamez 2021 [85]	Soccer	Personal archives from a leading ALS Unit, PubMed, specialized soccer websites, self-reports of patients in the media	Male population of Catalonia	Having played in the first, second and third tiers of the Spanish league between 1968 and 2013, and who developed ALS between 2000 and 2020	**	-	*	7/10780 players	–	SIR 2.11 (95% CI 0.85–4.37)
Mackay 2020 [82]	Soccer	Death certificates with ICD-9 and ICD-10 codes (primary and contributory cause) for MND from electronic health records	controls from the general population who were matched to the players based on sex, age, and degree of social deprivation	Former professional soccer players born before January 1, 1977	**	**	**	22/7676 players	17/23028 non-players	MND HR for death (95% CI) 4.33 (2.05–9.15) *p*: < 0.001
Pupillo 2020 [83]	Soccer	cases identified using different sources: Google, 2 published books, a recent scientific report	ISTAT mortality tables (since 1975)	Professional soccer players practicing in the period 1959–2000 (Panini almanacs)	**	*	*	34/23586 players	-	ALS overall SIR: 1.91 (95% CI 1.32–2.67) Subjects aged < 45 years SIR: 4.66 (95% CI 2.66–7.57) League A SIR: 5.69 (95%CI 2.73–10.47)
Nguyen 2019 [79]	American football baseball	Death certificates from 1979 to 2013 classified with ICD underlying (U) and underlying or contributing (U&C)	MLB cohort: from LBD of all players that appeared in ≥ 1 game at a professional level in 1871–2006	NFL cohort: from NIOSH consisting in players who played ≥ 1 season in 1959–1988	**	**	**	NFL U: 9/3419 U&C: 10/3419	MLB U: 3/2708 U&C: 3/2708	ALS HR (95%CI) U: 2.81 (0.75–10.51) U&C: 3.10 (0.84–11.38)
Janssen 2017 [77]	American football basketball swimming wrestling	3-step procedure: screening of medical index through REP tool, review of diagnoses, diagnosis confirmed by senior neurologist	Varsity athletes (football vs non-football) enrolled in the Mayo and Rochester High Schools in 1956 to 1970	Varsity activity in football, basketball, swimming or wrestling documented in the yearbooks	**	*	**	0/296 football players	0/190 non-football athletes	ALS IRR football: 0.00 (0.00–1.92) non-football: NA p NA
Case–control studies										
Filippini 2020 [81]	Any sport Soccer Volleyball Cycling Swimming	Recruited either at the Neurology Units of the study area or by contacting them by phone and/or mail Cases from 2 registries (ERRALS and PARALS) diagnosed with ‘definite’ and ‘probable’ ALS based on El Escorial revised criteria	National Health Service directory of the residents in the study provinces Random controls, matched by sex, age (± 5) and province of residence, from 4 populations	Having played a sport at a competitive level	**	**	-	Cases 95/230 Controls 135/230	-	Any sport Cases 10 vs controls 22 adj OR 0.50 (0.22–1.17) Soccer Cases 6 vs controls 6 adj OR 1.19 (0.35–4.02) Volleyball Cases 1 vs controls 4 adj OR 0.28 (0.03–2.64) Cycling Cases 1 vs controls 5 adj OR 0.31 (0.03–2.94) Swimming Cases 1 vs controls 1 adj OR 1.61 (0.09–27.33)

*MLB* US Major League Baseball, *NFL* National Football League, *SIR* standardized incidence ratio, *SMR* standardized mortality ratio, *HR* hazard ratio, *IRR* incidence rate ratio, *OR* odds ratio, *NA* not applicable, *CI* confidence interval, *U* underlying, *U&C* underlying and contributing

**Table 3:** Summary of results from studies reporting data on the frequency of MCI and dementias

Characteristics					Quality assessment			Summary of findings		
								*N*. of subjects		Results
Fist author, year	Sport	Source of cases	Reference population	Exposure	Selection	Comparability	Outcome/exposure	*n*. cases among the exposed/total exposed	*n*. cases among the reference group/total reference	Data ORs/HRs (95%CIs) or SIRs/PRs/SMRs/SPMRs
Cohort studies										
Russell 2021 [84]	Soccer	Hospital records, prescriptions and death certificates with ICD-9 and ICD-10 codes	Random sample of matched individuals (3:1 ratio) from the community health index database	Having been a soccer player in the Scottish League as documented in the archives of the Scottish Football Museum and individual league clubs	***	**	**	338/7676 players	312/23028 non-players	frequency of dementia (not otherwise specified) diagnosis HR 3.59 (95% CI 2.93–4.39) *p* < 0.001
Mackay 2020 [82]	Soccer	Electronic health records to obtain data on death certification and medications typically prescribed for the treatment of dementia ICD-9 and ICD-10 codes that were used to identify outcomes as death with neurodegenerative disease listed as the primary or a contributory cause	Controls from general population matched to players based on sex, age, and degree of social deprivation	Former professional soccer player born before January 1, 1977	**	**	**	Dementia 180/7676 players 178 non-players AD 64/7676 players non-AD dementia 121/7676 players	Dementia 178/23,028 non-players AD 47/23,018 non-players Non-AD dementia 133/23,018 non-players	dementia HR for Death (95% CI): 3.87 (2.86–5.24) *p*: < 0.001 AD HR for Death (95% CI): 5.07 (2.92–8.82) *p*: < 0.001 non-AD dementia HR for Death (95% CI): 3.48 (2.42–5.00) *p*: < 0.001
Nguyen 2019 [79]	American football baseball	Death certificates from 1979 to 2013 classified with ICD underlying (U) and underlying or contributing (U&C)	MLB cohort: from LBD of all players that appeared in ≥ 1 game at a professional level in 1871–2006	NFL cohort: from NIOSH consisting in players who played ≥ 1 season in 1959–1988	**	**	**	NFL U: 6/3419 players U&C: 16/3419 players	MLB U 7/2708 players U&C 10/2708 players	Dementia/AD HR (95%CI) U: 1.28 (0.41–4.07) U&C: 2.26 (0.99–5.17)
Willer 2018 [78]	American football hockey	Comprehensive neuro-cognitive assessment	Age-matched noncontact sport athlete controls	Retired professional hockey and football athletes (average age 56 years)	**	*	*	8/21 contact athletes	3/21 non-contact athletes	MCI frequency contact sport vs noncontact sport 8/21 vs 3/21 *p* = 0.08
Janssen 2017 [77]	American football basketball swimming wrestling	3-step procedure: screening of medical index through REP tool, review of diagnoses, diagnosis confirmed by senior neurologist	Varsity athletes (football vs non-football) enrolled in the Mayo and Rochester High Schools in 1956 to 1970	Varsity activity in football, basketball, swimming, or wrestling documented in the yearbooks	**	*	**	7/296 football players	5/190 non-football athletes	> 1 year of playing football: 4.99 (1.62–11.65) non-football: 3.24 (0.88–8.28) IRR: 1.54 (0.50–3.60), *p* = 0.26
Vann Jones 2013 [76]	Soccer	Test Your Memory, self-administered questionnaire	Frequency also compared to data from a large UK-based MCI prevalence study	Having been a soccer player from Former Player Associations (FPAs) of 4 professional football clubs in the UK	**	*	*	MCI—possible dementia 10/92 soccer players	–	Overall MCI/dementia 10/92 MCI: 8/92 dementia: 2/92 No significant difference in cognitive impairment prevalence between ex-professional footballers and a large sample of men in Wales
Lehman 2012 [74]	American football	Death certificates with ICD code for AD in effect at death	US male mortality rates (1960–2007) for 119 cause of death categories	Having played in the National Football League for at least 5 seasons between 1959 and 1988	****	**	*	U: 2/3439 players C: 7/3439	–	AD SMR-U: 1.80 (0.22–6.50) SMR-U&C: 3.86 (1.55–7.95)
Savica 2012 [75]	American football	Medical records confirmed by a neurologist	Having been a band, glee club, or choir member as for the yearbooks of the only 2 high schools in Rochester in 1946–1956 Expected incidence calculated based on previously published rates for that area	Having been a football player as for the yearbooks of the only 2 high schools in Rochester in 1946–1956	***	**	**	13/438players	2/140 non-players	Dementia HR (95%CI) 1.58 (0.36–7.01) (*p* = 0.55) SIR (95%CI) observed vs expected players: 0.72 (0.38–1.23) non-players: 0.47 (0.05–1.68)
Guskiewicz 2005 [72]	American football	AD diagnosed by clinicians MCI diagnosed by clinician, self-reported or reported by informant	General US Population (AD)	Members of the National Football League Retired Player’s Association	**	*	*	AD 33/2252 MCI diagnosed 22/758 MCI reported by informant 77/758	–	AD 33/2252 overall age-adjusted prevalence ratio 1.37 (95% CI 0.98–1.56) higher in (≤ 70 years) 22 diagnosed MCI and 77 memory impairment
Schulte 1996 [43]	Athletes (any)	Death certificates with ICD-9 code for NDs	Deaths for all causes 1982–1991, and proportion of NDs deaths in all occupations	occupation and industry coded as “writers, artists, entertainers, and athletes” according to the 1980 Bureau of the Census classification	****	**	*	265 deaths (white female writers, artists, entertainers, or athletes)/130420 total deaths	–	AD PMR: 135 (95% CI 111–162)
CASE–CONTROL STUDIES										
Park 2005 [44]	Athletes (any) dancers	Death certificates with ICD-9 code for NDs	Deaths for all other causes (excluding CNS neurologic, degenerative, and neoplasms of brain, lymphatic, or hematopoietic tissue)	Certificates, coded according to the 1980 Bureau of Census (BOC) system	***	**	**	Pre-senile dementia 27,374/2,614,346 AD 47,783/2,614,346 (total n° of deaths 2,614,346)		MOR (95%CI) presenile dementia: 0.93 (0.46–1.66) AD: 1.46 (0.96–2.10)

*MLB* US Major League Baseball, *LBD* Lahman Baseball Database, *HR* hazard ratio, *IRR* incidence rate ratio, *SMR* standardized mortality ratio, *SIR* standardized incidence ratio, *PMR* proportionate mortality ratio, *MOR* mortality odds ratio, *CI* confidence interval, *U* underlying, *U&C* underlying and contributing

**Table 4:** Summary of results from studies reporting data on the frequency of PD

Characteristics					Quality assessment			Summary of findings		
								*N*. of subjects		Results
Fist author, year	Sport	Source of cases	Reference population	Exposure	Selection	Comparability	Outcome/exposure	*n*. cases among the exposed/total exposed	*n*. cases among the reference group/total reference	Data ORs/HRs (95%CIs) or SIRs/PRs/SMRs/SPMRs
Cohort studies										
Russell 2021 [84]	Soccer	Hospital records, prescriptions and death certificates with ICD-9 and ICD-10 codes	Random sample of matched individuals (3:1 ratio) from the community health index database	Having been a soccer player in the Scottish League as documented in the archives of the Scottish Football Museum and individual league clubs	***	**	**	28/7676 players	45/23028 non players	frequency of PD diagnosis HR 2.09 (95% CI 1.20–3.61) *p* = 0.009
Mackay 2020 [82]	Soccer	Death certificates with ICD-9 and ICD-10 codes (primary and contributory cause) for PD from electronic health records	Controls from general population matched to the players according to sex, age, and degree of social deprivation	Former professional soccer players born before January 1, 1977	**	**	**	28/7676 players	44/23028 non players	PD HR for death (95% CI): 2.15 (1.17–3.96) *p* = 0.01
Nguyen 2019 [79]	American football baseball	Death certificates from 1979 to 2013 classified with ICD underlying (U) and underlying or contributing (U&C)	MLB cohort: from LBD of all players that appeared in ≥ 1 game at a professional level in 1871–2006	NFL cohort: from NIOSH consisting in players who played ≥ 1 season in 1959–1988	**	**	**	NFL U: 3/2708 U&C: 14/3419	MLB U: 3/2708 U&C: 5/2708	HR (95%CI) U: 3.08 (0.76–12.42) U&C: 3.45 (1.21–9.83)
Janssen 2017 [77]	American football basketball swimming wrestling	3-step procedure: screening of medical index through REP tool, review of diagnoses, Diagnosis confirmed by senior neurologist	Varsity athletes (football vs non-football) enrolled in the Mayo and Rochester High Schools in 1956 to 1970	Varsity activity in football, basketball, swimming or wrestling documented in the yearbooks	**	*	**	3/296 football players	3/190 non-football athletes	Incidence rate ratio (IRR) parkinsonism (95% CI) football: 2.08 (0.57–5.32) Non-football:0.86 (0.23–2.19) *p* > 0.99
Lehman 2012 [74]	American football	Death certificates with ICD code for PD in effect at death	US male mortality rates (1960–2007) for 119 cause of death categories	Having played in the National Football League for at least 5 seasons between 1959 and 1988	****	**	*	U: 2/3439 players C: 3/3439 players	–	PD SMR-U: 2.14 (0.26–7.75) SMR-U&C: 4.31 (1.73–8.87)
Savica 2012 [75]	American football	Medical records confirmed by a neurologist	Having been a band, glee club or choir member as for the yearbooks of the only 2 high schools in Rochester in 1946–1956 Expected incidence calculated based on previously published rates for that area	Having been a football player as for the yearbooks of the only 2 high schools in Rochester in 1946–1956	***	**	**	10/438 players	5/140 non-players	HR (95%CI) 0.48 (0.17–1.42) (*p* = 0.19) SIR (95%CI) observed vs expected players: 2.36 (1.13–4.34) non-players: 4.94 (1.61–11.51)
Lolekha, 2010 [73]	Boxing	2-phase procedure: screening with validated Tanner’s questionnaire and Sevillano weighted score and diagnosis with UKPDSBB clinical diagnostic criteria	Several populations	Registered retired boxers from the Sports Authority of Thailand, the Boxer’s Club of Thailand and the old Thai Boxing Foundation	***	*	–	8/704 boxers	–	8/704 Parkinsonism (5 PD, 1 PSP, 2 vascular parkinsonism) crude prevalence: 0.71% (95% CI 0.09–1.33) comparable to general populations
Schulte 1996 [43]	Athletes (any)	Death certificates with ICD-9 code for NDs	Deaths for all causes 1982–1991, and proportion of NDs deaths in all occupations	Occupation and industry coded as “writers, artists, entertainers, and athletes” according to the 1980 Bureau of the Census classification	****	**	*	128 deaths (white female writers, artists, entertainer, or athletes)/130420 total deaths		PMR: 128 (95% CI 107–152)
Case–control studies										
Park, 2005 [44]	Athletes (any) dancers	Death certificates with ICD-9 code for NDs	Deaths for all other causes (excluding CNS neurologic, degenerative, and neoplasms of brain, lymphatic or hematopoietic tissue)	Certificates, coded according to the 1980 Bureau of Census (BOC) system	***	**	**	33,678 PD total deaths: 2,614,346	–	MOR 1.26 (95% CI 0.76–1.97)

*MLB* US Major League Baseball, *LBD* Lahman Baseball Database, *HR* hazard ratio, *IRR* incidence rate ratio, *SMR* standardized mortality ratio, *SIR* standardized incidence ratio, *PMR* proportionate mortality ratio, *MOR* mortality odds ratio, *CI* confidence interval, *U* underlying, *U&C* underlying and contributing, *PSP* progressive supranuclear palsy

**Table 5:** Summary of results from studies reporting data on the frequency of CTE

Characteristics					Quality assessment			Summary of findings		
								*N*. of subjects		Results
Fist author, year	Sport	Source of cases	Reference population	Exposure	Selection	Comparability	Outcome/exposure	*n*. cases among the exposed/total exposed	*n.* cases among the reference group/total reference	Data ORs/HRs (95%CIs) or SIRs/PRs/SMRs/SPMRs
Cohort studies										
Bieniek 2019 [80]	Baseball Basketball Boxing American football Hockey Soccer Wrestling	Mayo Clinic Tissue Registry	consecutive autopsies from the Mayo Clinic Tissue Registry with available information on sport participation	information on sport participation and participation age/level/duration taken from high school yearbooks or obituaries	***	–	***	300 athletes CTE positive:r 15 athletes CTE features: 12	450 non-athletes CTE positive: 6 CTE features: 9	OR* for combined CTE pathology (males) adj for age at death, baseball, boxing, and American football American football beyond high school OR = 13.23 (4.23, 41.36) *p* < 0.001 OR for combined CTE positive (CTE +) (males) adj for boxing and American football boxing OR = 10.29 (1.79, 59.28) *p* = 0.009 American football OR = 2.87 (1.17, 7.01) *p* = 0.021 American football beyond high school OR = 6.84 (1.76, 26.66) *p* = 0.0056

*OR* odds ratio

*Only adjusted data reporting statistically significant differences have been reported

### Methodological quality

The overall methodological quality of included studies was moderate. The highest score achieved was 7, while the lowest score was 3. Five cohort studies achieved a score of 7 [43, 74, 75, 84, 86] while 4 studies, 3 cohort [79–81] and 1 case–control [44] were assigned 6 stars, 2 cohort studies were assigned 5 stars [73, 77], 5 studies, 4 cohort [72, 76, 78, 83] and 1 case–control [81] were assigned 4 stars, and 1 cohort study [85] was assigned 3 stars.

#### Selection

Only two cohort studies reached 4 stars, which is the highest possible score, in this domain, while most studies were scored 2 stars. All included cohort studies enrolled professional or varsity athletes from secure records (e.g., sports leagues, yearbooks), thus the enrolled exposed cohort is often adequately defined and representative, and data on exposure are drawn from secure sources. However, the unexposed cohort is often enrolled from different sources from the exposed cohort, and most of the studies did not or could not (e.g., studies using census data) provide information on the absence of the outcome at the beginning of the study.

As for the two case–control studies, cases were enrolled through either registries or death certificates. Therefore, samples were representative, but the definition of cases did not have an independent validation. In both studies, controls were enrolled from the community, as they were either randomly selected from general population or selected among death certificates due to other non-degenerative causes. However, both studies could not and did not ascertain the absence of the outcome at the start of the study.

#### Comparability

Most studies reached 2 stars (*n* = 8), which is the highest possible score, in this domain, while 6 studies were assigned 1 star, and 2 studies had no stars [73, 80]. Most studies were either matched or had the analysis adjusted for age [72, 76–78, 83] and/or for other variables. In some studies, matched controls were selected from general population, while in some other studies controls were either other athletes [77–79] or students from the same schools [75]. In case–control studies, controls were either matched for sex, age, and province of residence [81] or selected based on the availability of enough information for adjusting all analyses [44].

#### Exposure/outcome

Only 2 cohort studies reached 3 stars in this domain, which is the highest possible score [80, 86] while 6 studies had 2 stars, 6 studies had 1 star, and 1 case–control study and 1 cohort study had no stars [81, 85].

Most of the cohort studies used structured information from death certificates, clinical records, registries, or record-linkage. Of the five remaining studies, 2 studies obtained data on the outcome by clinicians [72, 78] 2 from questionnaires [73, 77] and 2 only from databases or search engines (i.e., PubMed, Google) or books and magazines [83, 85]. Using secure sources such as hospital records and certificates or using an independent validation of the diagnosis to confirm the outcome can minimize the potential for inaccurate information. However, even if death certificates are an extremely valuable source of data, their accuracy in reporting deaths due to NDs/NCDs can be inaccurate. In fact, deaths due to the considered NDs/NCDs were shown to be underreported as primary cause of death [87–94] and studies on mortality are often of poor methodological quality [95].

Moreover, most of the included studies used, for all the considered outcomes, widely heterogeneous diagnostic criteria. Using a heterogeneous set of criteria prevent the direct comparison of data, as different criteria can lead to different prevalence and incidence rates [96–98].

Another issue was the length of the follow-up, as at least part of the included sample in most studies using administrative data had a follow-up length insufficient to observe the considered outcome.

As for case–control studies, one [44] had a secure source for ascertaining the exposure, while the other [81] used an unstructured questionnaire and did not use the same method for cases and controls. Moreover, neither reported the response rate for both cases and controls. Moreover, most of the studies did not report the number of participants lost to follow-up.

### Qualitative analysis

Results from the included studies were reported according to outcome. A summary of the characteristics and results from each study are reported in Tables 1, 2, 3, 4, and 5.

#### All NDs/NCDs

Only 5 cohort studies reported data on the overall risk of any type of NDs and/or NCDs in former professional/varsity athletes. Specifically, 3 of the 5 cohort studies reported the risk of NDs/NCDs in either former professional American football players [74, 79] or former varsity American football players [77] while the remaining 2 studies reported the risk of NDs/NCDs in former professional soccer players [82, 84]. Both studies on professional American football players enrolled former players from the National Football League (NFL) and used death certificates to calculate their risk of death due to NDs/NCDs as underlying (U) and/or contributing (C) cause of death. The studies reported an overall higher standardized mortality ratio (SMR) due to NDs/NCDs in former NFL players (SMR-U 2.83, 95%CI 1.36–5.21; SMR-U&C 3.26, 95%CI 1.90–5.22) [74] with a specifically higher risk of NDs/NCDs in former NFL players compared to baseball players (HR-U 2.07, 95%CI 1.01–4.23; HR-U&C 2.99, 95%CI 1.64–5.45) [79]. A significantly higher risk of death due to NDs/NCDs was also observed in the 2 studies enrolling former professional soccer players, with HRs ranging from 3.53 to 4.10 (HR-U 4.10, 95%CI 2.88–5.83; HR-U&C 3.53, 95%CI 2.72–4.57; HR 3.66, 95%CI 2.88–4.65) [82, 84]. No risk measure was calculated for former varsity athletes, but the study observed that 3% of the enrolled football athletes reported a diagnosis of NDs/NCDs compared to 4% of the athletes enrolled in the basketball, swimming, or wrestling teams [77].

#### ALS or MND

The previous review [23] on the risk of ALS and MND in former professional and varsity athletes reported a significantly higher frequency of ALS and MND in professional soccer and American football players, and a slightly increased risk of ALS in varsity athletes. The update of the SR allowed to identify 6 new cohort studies [77, 79, 82–85] and 1 new case–control study [81]. When considering professional athletes, 5 large cohort studies reported a significantly higher risk of ALS and MND in former soccer and American football players (MND: HR 3.52, *p* < 0.001; HR 4.33, *p* < 0.001; ALS: SIR 1.91; ALS: SIR 2.11; ALS: SIR 3.59) [82–86] compared to either general population [79–82] or matched controls [78]. Two studies specifically reported a significantly higher risk in subjects aged < 45 (SIR: 4.66, 95% CI 2.66–7.57) years and in athletes playing in the top league (SIR 5.69, 95%CI 2.73–10.47) [83] and in athletes having longer careers (OR 1.2, 95% CI 1.1–1.3) [86]. One study also reported a higher risk of death due to ALS compared to general population (SMR 3.94) [86]. Another cohort study used death certificates and did not report a higher risk of death due to ALS in former NFL players compared to baseball players [79]. The last cohort study enrolled varsity athletes from yearbooks and compared the frequency of ALS (i.e., healthcare information system) between football and non-football player, reporting no cases of ALS during its follow-up period [77]. The only, relatively small, case–control study enrolled cases from 2 registries and matched controls from general population and did not observe a significantly higher risk of ALS in athletes (i.e., any type of sport) compared to non-athletes [81].

#### Dementia and/or MCI

Ten cohort studies [43, 72, 74–79, 82, 84] and 1 case–control study [44] reported data on the risk of dementia in former professional and/or varsity athletes. Seven cohort studies [72, 74–76, 78, 79, 82, 83] reported information on the frequency of dementias or MCI in former professional athletes. When considering former soccer players, 2 large cohort studies enrolled participants from museum and league club archives and structured healthcare information systems, reporting a higher frequency of dementia (HR 3.59, *p* < 0.001; HR 3.87, *p*: < 0.001) and AD (HR 5.07, *p*: < 0.001) in former soccer players compared to general population [82, 84]. A small study enrolled former soccer players from 4 soccer clubs and did not report an increased risk NCDs as tested with a self-administered questionnaire in former players, irrespective of role and length of career, compared to general population [76]. As for American football, 3 cohort studies reported a slightly higher risk of AD in former NFL players [72, 74, 79]. The first two studies enrolled former NFL player from retired players associations and reported respectively a higher risk of AD compared to general US population (prevalence ratio 1.37) [72], and of death due to AD compared to US male mortality rates (SMR-U 1.80, SMR-U&C 3.86) [74], with the second reporting a significantly higher risk in athletes who played in speed positions (SMR 6.02) [74]. The third cohort study used data from the National Institute of Safety and Health (NIOSH) and reported a slightly, non-significantly, higher risk of death due to dementia/AD (i.e., death certificates) in former NFL players compared to baseball players (HR-U 1.28, HR-U&C 2.26) [79]. When considering varsity athletes, 2 cohort study enrolled former athletes from yearbooks [75, 77]. The first reported no significant differences in the frequency of dementia and MCI in former football players compared to non-football players [77], while the second reported no significant increase in the risk of dementia in former football players compared to participants in non-sport-related clubs [75]. Two further studies, 1 cohort, and 1 case–control study, investigated the association between occupational exposure to professional sports and the risk of death due to NCDs using census data and death certificates [43, 44]. The cohort study reported a significantly higher risk of AD in subjects whose occupation was coded as “writers, artists, entertainers, and athletes” (PMR: 135 (95% CI 111–162) [43], while the case–control study reported no significant increase on risk of AD or pre-senile dementia in subjects whose occupation was coded as “athlete or dancer” [44].

When considering MCI, a small cohort study enrolled participants from alumni associations and reported a slightly, non-significantly, higher risk of MCI (i.e., comprehensive neurocognitive assessment) in former NFL and hokey players compared to former non-contact sport athletes [78]. A small cohort study reported no increased risk of MCI based on a self-administered test in former soccer players from former player associations compared to general population (i.e., data from a large population study on MCI) [76]. However, one study on former NFL players from retired NFL players associations reported a correlation between MCI or memory impairment (MI) (i.e., diagnosed, self-reported, reported by informant) and self-reported recurrent concussion (MCI *p* = 0.02; self-reported MI *p* = 0.001; spouse/relative-reported MI *p* = 0.04) [72].

#### Parkinson's disease (PD)

Eight cohort studies [43, 73–77, 79, 82, 84] and 1 case–control study [44] reported data on the risk of PD in professional and varsity athletes. As for professional athletes, 2 cohort studies used data from archives of museums or clubs and hospital records, prescriptions, and death certificates, and reported a significantly higher frequency of PD in former soccer players (HR 2.09, *p* = 0.009; HR 2.15, *p* = 0.01) [82, 84]. Two further cohort studies used data from information systems (e.g., NIOSH database), and reported a higher risk of death due to PD (i.e., death certificates) in former American football players compared to general population (SMR-U&C 4.31) [74] or to former baseball players (HR-U&C 3.45) [79], with no significant difference between athletes playing in speed vs non-speed position [74]. One cohort study in a relatively limited sample of former Thai boxers enrolled from registers of former boxers (i.e., sports authority, boxers’ clubs, and Thai boxing foundation) reported a prevalence of PD and Parkinsonism, as assessed through a validated scales and questionnaires, comparable to the prevalence observed in several Asian general populations [73]. Two further studies, 1 cohort and 1 case–control, investigated the association between occupational exposure to professional sports and the risk of death due to PD using census data and death certificates [43, 44]. Of these, 1 reported a higher risk of death due to PD in the category of subjects whose occupation was categorized as “Writers, artists, entertainers, and athletes” (PMR: 128) [43], while the other reported no significantly increased risk of PD in subjects whose previous occupation was classified as “athlete” [44]. When considering varsity athletes, 2 studies enrolled former athletes from yearbooks and did not report a higher risk of PD or Parkinsonism in former American football players compared to either members of other non-sport-related clubs [75] or to non-football athletes [77].

#### Chronic traumatic encephalopathy (CTE)

Only one study reported data on the risk of CTE in former competitive athletes. The study enrolled participants from a brain tissue bank, ascertaining exposure to previous competitive sport participation (e.g., varsity, professional) through obituaries or school yearbooks. In this study, participants were classified as CTE positive (CTE +) in case of pathology fitting with all aspects of the consensus criteria for CTE neuropathology, and as “features of CTE” in case of multiple lesions suggestive of CTE pathology without being consistent with all parts of the criteria verbatim. CTE + and features of CTE were classified together as “combined CTE patology”. CTE + was also evaluated separately as secondary outcome. The study reported a significantly higher risk of CTE in participants who played American football both at during (CTE + OR = 2.87, *p* = 0.021) and after high school (CTE pathology OR = 13.23, *p* < 0.001, CTE + OR = 6.84, *p* = 0.0056), and in former boxers (CTE + OR = 10.29, *p* = 0.009) [80].

### Quantitative analysis

No meta-analyses were carried out due to the clinical, methodological, and statistical heterogeneity of the included studies. The main reasons that prevented the cumulative analysis of data were due to the study designs being different, and their methodological quality not being homogeneous. As for clinical heterogeneity, the definition and source of both the reference population and the cases were widely variable across studies. Moreover, the investigated cohorts of athletes were mostly the same cohorts observed in different time-periods, as in the case of the two large cohort studies on mortality due to NDs among Scottish professional soccer players.

## Discussion

Overall, the present systematic review reported a higher frequency of NDs/NCDs in former professional athletes. Specifically, published studies showed an increased frequency of any type of NDs/NCDs in former soccer players [82, 84] and American football players [74] even when compared to other non-contact sport athletes [79]. When considering specific NDs and NCDs, data from the update of the previous SRs confirmed a higher frequency of ALS or MND in former soccer players [82–85]. Gathered data also reported a significantly higher risk of dementia and AD in former soccer players [82, 84] and a higher risk of MCI in former American football players, mainly associated to a higher risk of concussion [72]. Moreover, data also showed a significantly higher risk of PD in former soccer and American football players [74, 75, 79, 82, 84] and a significantly higher risk of CTE in former boxers and in former American football players [80].

Some studies reported either non-significant results or no increase in risk in some categories of athletes. However, most of these studies were of low or relatively low methodological quality and included very small or relatively small samples defining cases based on self-reports or non-standardized/validated tools. Moreover, some studies only compared different types of sports (i.e., contact vs non-contact, football vs non-football). The only large and high-quality study [44] reporting no difference in the mortality rate for any of the considered NDs and NCDs considered as exposed participants classified as “athlete/dancer” based on the occupational census, which is a wider definition that could lead to potentially underestimating results. Most of the included studies attempted to take into consideration potential confounding factors by either matching non exposed subjects or controls by at least the main characteristics (e.g., gender, age, ethnicity), with some of them also stratifying for potential interacting factors such as length of career and type of sport or position/role played. However, the factors considered for adjustment where heterogeneous across some of the included studies, and most studies only include male professional players due to the availability of data. This further affected the direct comparison of data. Previous reviews suggested that the higher risk of NDs observed in some specific categories of former athletes could be sport specific. However, no studies on sports other than soccer, American football and marginally boxing, were available. Therefore, conclusion can only be based on available studies, as no data are currently available on different sports. Further large studies should be performed on other sports, mainly in other contact sports (e.g., boxing, martial arts) and sports requiring running/speed roles (e.g., rugby, hokey, etc.), should be carried out, as these types of sports resulted to be associated with the highest risk of NDs and NCDs.

As for ALS and MND, our results confirmed the higher risk observed in formed soccer and American football player reported in the previously published SR [23]. Included studies reported an increased risk of ALS in soccer and American football players also confirmed an earlier onset of ALS in soccer players, and a higher frequency of ALS in players with a longer career, in specific positions or roles, and playing in main leagues [82–84, 86]. Therefore, based on results from the previous SR and the present update, soccer and American football players seem to be at risk of a specific, more severe, type of ALS or MND, with earlier onset (< 45 years) and shorter survival.

Our data also showed an increased risk of dementia, AD and MCI in former soccer and American football players. This increase in the frequency of these conditions was linked, in some studies, to an increased exposure to brain injuries and concussions, specifically in former American football players. In this last category of athletes, a higher frequency of CTE was also observed. As expected, the risk of CTE resulted the highest in former boxers, who were among the first categories of athletes in whom the formerly called “punch drunk syndrome” and “dementia pugilistica” were investigated [99, 100].

Moreover, a higher risk of PD and Parkinsonism was observed in former soccer and American football players, which was not linked to specific roles or positions. This increased frequency of PD, as for dementia and cognitive decline, was also generally linked to an increased exposure to brain injury and trauma.

On this basis, our results seem to suggest that competitive athletes, and specifically those participating in contact sports, are exposed to a higher risk of trauma, concussion, and subsequent brain injuries, thus leading to a higher risk of developing cognitive outcomes or PD/Parkinsonism later in life. However, professional athletes appear to also be at a higher risk of what seems to be a specific form of ALS/MND. Based on our data and data from our previous SR, in fact, the type of ALS/MND observed in former soccer players seem to have a much earlier, bulbar onset, and a much shorter survival. This increased risk has been linked to several possible genetic and environmental risk factors. Several gene variants are associated with a susceptibility to ALS/MND, and when considering environmental risk factors, the exposure to heavy metals, trace elements, solvents, and other volatile organic compounds (e.g., pesticides and radiation) or toxins (e.g., cyanobacteria), has been linked to a higher risk of developing this condition. As for some life-style factors, the widespread use or abuse of branched-chain amino acids in professional athletes has been hypothesized to be potentially linked to a higher risk of ALS/MND [28]. This led to the specific hypothesis that professional athletes might share a specific phenotype linked to both a better predisposition to high-level sport and a risk of developing ALS/MND. However, all these genetic, environmental, and lifestyle risk factor are shared risk factors for all the considered NDs and NCDs. The exposure to some pesticides, including organophosphorus compounds and carbamates, in fact, has been reported to be associated not only to an increased risk of ALS, but also to a higher risk of AD and PD [101]. Several other compounds, such as solvents and neurotoxic agents, have also been proven to be shared risk factors for ALS and other NDs and NCDs, in particular PD [102]. Moreover, several polymorphisms and gene variants have been reported to be associated to a higher frequency of specific NDs, and some have also been investigated as involved in the epigenetic mechanisms leading to the onset of the disease [103–106]. The exposure to several lifestyle factors, including smoking, physical activity, and dietary habits along with the use/abuse of some dietary supplements, have also been proven to have a role in the etiological mechanisms of some NDs and NCDs. The evident interlinking and interaction among these factors, and their being shared risk factors for several NDs and NCDs underline the need to start considering neurodegeneration as a process starting with an early, subclinical, phase, which might be common to all NDs. The neurodegenerative process in NDs, in fact, has been proven to start several decades before the onset of symptoms [107]. This long latent period, along with the shared association between several risk factors and some specific NDs suggests that this early process might be common to different NDs and only in a subsequent phase cause specific symptoms according to a subjective susceptibility and specific interactions between risk factors [3, 108]. Thus, based on this hypothesis, research should focus on investigating the interacting role of genetic, environmental, and lifestyle risk factors in this early phase of neurodegeneration to explore the possibility of defining potential preventive strategies [106]. Moreover, former professional athletes should be further investigated, as these risk factors and their interactions, as well as the risk of some specific NDs, appear to be significantly higher in this population. Studies on this population should also aim at clarifying whether the risk might be specific to some type of sports or roles, and whether the clinical characteristics of the NDs are typical of this population.

Our SR has some limitations. The main limitation is the relatively small number of included studies. Studies on this topic are relatively few and are limited to a very small number of sports, mainly soccer and American football. This limits the generalizability of results and the reliability of results. The small number of published studies might be due to the difficulty of investigating a relatively rare exposure as having been a professional athlete, and the subsequent difficulty of enrolling large samples of professional athletes. Moreover, when considering ALS, the difficulty of carrying out large studies might also be due to the rarity of the disease, which would require very large samples. Furthermore, NDs and NCDs are diseases whose typical onset is later in life, thus longitudinal studies require covering very long observation periods. Therefore, prospective studies are almost unfeasible, and retrospective studies might be very difficult to carry out in absence of effective and efficient informative systems and databases. However, the use of data from information systems, such as death certificates and hospital records, largely underestimates the frequency of NDs and NCDs, thus limiting the quality of studies, as does the use, inevitable in these systems, of different diagnostic criteria. A further limitation, due to the above-mentioned difficulties, is that the considered cohorts of athletes should be intended as mostly the same cohorts observed in different time periods. This prevents the comparison and the cumulative analysis of data, allowing only to observe a possible trend in the risk of NDs and NCDs over time within the same cohort. Further high-quality studies including athletes participating in different sports would therefore be extremely useful to assess whether the observed higher risk might be sport-specific or shared with other types of sport, and to further investigate possible characteristic or risk factors suggesting specific pathophysiological mechanisms explaining the observed increase in the risk of NDs and NCDs.

## Data Availability

The present SR was not registered. The datasets used and/or analyzed during the current study are available from the corresponding author on reasonable request.
